# Pregnancy after heart and kidney transplantation: a case report

**DOI:** 10.1016/j.jhlto.2024.100059

**Published:** 2024-01-23

**Authors:** Farnaz Ahmadi, Farah Naghashzadeh, Zargham Hossein Ahmadi, Babak Sharif-Kashani, Seyed Mohammad Reza Nejatollahi, Shakiba Khodadad, Seyed Mohsen Mirhossein, Zahra Ansari Aval, Alireza Jahangirifard, Leila Saliminejad, Sourena Sharif-Kashani, Shadi Shafaghi, Sima Noorali

**Affiliations:** a Lung Transplantation Research Center, National Research Institute of Tuberculosis and Lung Diseases (NRITLD), Shahid Beheshti University of Medical Sciences, Tehran, Iran; b Tobacco Prevention and Control Research Center, National Research Institute of Tuberculosis and Lung Diseases (NRITLD), Shahid Beheshti University of Medical Sciences, Tehran, Iran; c Hepato-Pancreato-Biliary and Transplant Surgery, Masih Daneshvari Hospital, Shahid Beheshti University of Medical Sciences, Tehran, Iran; d Department of Gynecology Oncology, Erfan Hospital, Tehran, Iran; e Cardiovascular Research Center, Department of Cardiovascular Surgery, Shahid Beheshti University of Medical Sciences, Tehran, Iran; f Tracheal Diseases Research Center, NRITLD, Shahid Beheshti University of Medical Sciences, Tehran, Iran; g School of Medicine, Tehran University of Medical Sciences, Tehran, Iran

**Keywords:** pregnancy, heart, lung, transplantation, combined transplantation, post-transplantation

## Abstract

**Background:**

The risk of pregnancy-associated complications is considerably elevated in transplant recipients compared to the general population, and it should be managed aggressively to preserve the allograft function and avoid complications. In the present case report, we described a 24-year-old woman with a transplanted heart and kidney who got pregnant after 6 years.

**Case presentation:**

A 24-year-old female who was diagnosed with congenital dilated cardiomyopathy and end-stage renal disease due to reflux nephropathy underwent combined heart and kidney transplantation. In her follow-up, she experienced pregnancy after 6 years. Afterward, a multidisciplinary team of different specialists monitored the pregnancy. After 40 weeks of pregnancy, a cesarean section was performed with obtaining a live female sex newborn with a weight of 2450 g, height of 50 cm, and Apgar score of 9, without any congenital malformations. In the follow-up, after 5 years, normal growth and development without any malformations and congenital disorders were observed.

**Conclusion:**

The successful outcome of pregnancy in this woman could be due to the proper cooperation between the medical team and patient, and also the appropriate timing of pregnancy.

## Background

Pregnancy after heart or kidney transplantation is identified as a high-risk condition for maternal and fetal outcomes, particularly due to the unavoidable administration of immunosuppressive agents.^1^ The risk of pregnancy-associated complications is considerably elevated in transplant recipients compared to the general population, including low birth weight, gestational diabetes, preeclampsia, prematurity, cesarean section, fetal growth restriction, and intrauterine fetal death. In fact, the complications are divided into 3 general categories: transplant-related maternal complications, maternal complications unrelated to transplantation, and fetal complications. Therefore, pregnancy in transplant recipients should be managed aggressively to preserve the allograft function, maintain normal metabolic processes, and avoid complications.^2^, ^3^

Although post-transplant pregnancy is usually discouraged by physicians due to concerns regarding potential risks to the fetus and the mother, successful pregnancy outcomes have been documented among recipients of transplants.^4^ The first document of a successful pregnancy in a heart transplant recipient was published in 1988, and the first pregnancy with acceptable clinical outcomes in a kidney transplant recipient was described in 1958.^3^

Unlike several life-threatening diseases, such as pulmonary hypertension, organ transplantation is not a contraindication for pregnancy.^5^ However, as far as our knowledge, there is limited data regarding pregnancy after combined heart and kidney transplantation. In fact, little is yet known surrounding maternal and fetal outcomes of pregnancy after combined heart and kidney transplantation. In addition, the long-term development, growth, and complications of infants born to these transplanted women is a topic less investigated.^6^

In the present case report, we describe a 24-year-old woman with a transplanted heart and kidney who experienced an uneventful pregnancy and labor and good 5-year outcomes for the infant and mother.

### Case presentation

A 24-year-old female who was diagnosed with a form of congenital dilated cardiomyopathy and end-stage renal disease due to reflux nephropathy underwent combined heart and kidney transplantation. The patient has experienced 5 phases, respectively, including pretransplant organ failure, transplantation, post-transplantation, pregnancy, and postpartum phase.

The initial immunosuppressive therapy included mycophenolate mofetil, tacrolimus, and prednisolone. The first echocardiography after transplantation showed a 60% ejection fraction. The first endomyocardial biopsy reported mild-degree inflammation with no eosinophilic infiltration, myocyte necrosis, venulitis, or interstitial hemorrhage. The patient was discharged after 3 weeks in good general condition. The contraceptive method was pharmacological, and after the procedure, she continued by combining rhythm and barrier methods simultaneously.

Six years after simultaneous heart-kidney trasplantation, an ultrasound was performed due to amenorrhea which confirmed a 2-week intrauterine pregnancy. Afterward, a multidisciplinary team of different specialists monitored the pregnancy, including a cardiologist, obstetrician-gynecologist, nephrologist, infectious disease specialist, and anesthesiologist. During pregnancy, mycophenolate mofetil was stopped and azathioprine was initiated in addition to the necessary supplements. Tacrolimus levels, blood urea nitrogen, creatinine, complete blood count, and echocardiography were conducted each trimester and then every 3 weeks in the last 2 months. An obstetrician controlled gestational factors every month till the 28th week, twice a month till the 36th week, and every week until delivery. The first echocardiography after pregnancy showed the normal function of the cardiac allograft with 55% left ventricular ejection fraction (LVEF). During gestational time, there were no complications, such as gestational diabetes, infection, or preeclampsia.

After 40 weeks of pregnancy, a cesarean section was performed, and a live female sex newborn weighing 2450 g, 50 cm in height, and an Apgar score of 9 without any congenital malformations was born. The patient was recommended to avoid breastfeeding the infant because of the risk of immunosuppressive drug excretion in breast milk.

Forty days after delivery, the patient was visited, and there was no complication with allografts or rejection. The second echocardiography after delivery was normal with LVEF = 60%; the endomyocardial biopsy showed no rejection, which is classified as grade 0R according to the International Society for Heart and Lung Transplantation guidelines; and medication after delivery included mycophenolate mofetil and tacrolimus. In the follow-up, after 5 years, normal growth and development without any malformations or congenital disorders (especially metabolic diseases) were observed.

## Discussion

Pregnancy causes physiologic changes in systemic hemodynamics, which can lead to several hemodynamic and immunologic complications in pregnancy after solid organ transplantation, resulting in an increased risk of graft rejection, therapeutic abortions, fetal growth restriction, fewer live births, and preterm delivery.^7^ In addition, hyperemesis gravidarum in post-transplant pregnant women is a serious issue since immunosuppressants may not be absorbed normally, increasing the risk of rejection.^3^ On the other side, all these immunosuppressive drugs can cross the placental barrier and enter the fetal circulation.^8^ The treatment necessary to control the disease during pregnancy and avoid fetal risks is not a minor issue, and currently, it represents a real challenge.^9^

Some studies reported an approximately 15% to 20% risk of spontaneous abortion in the postheart transplant pregnancy.^10^ However, some researchers noted higher percentages of spontaneous abortions related to the administration of immunosuppressant agents.^11^, ^12^ Recent studies indicated that kidney-transplanted pregnant women had elevated serum creatinine after pregnancy, confirming that pregnancy can adversely affect graft function.^13^

Thus, complete preconception counseling is essential to ensure a healthy pregnancy, manage maternal complications, and optimal birth outcomes. Several items should be considered, such as the underlying cause of heart failure, the timing of pregnancy, the safety of immunosuppressants, and comorbid conditions. During the pregnancy, the transplanted mother should also be monitored and closely followed by the expert team of specialists. The main target is maintaining alloimmunity quiescence during pregnancy and improving the outcome of the birth after delivery.^6^

According to previous studies, one of the most determinant factors in a successful pregnancy is the timing of the pregnancy. In this regard, Rose et al assessed allograft condition and pregnancy outcome in the first 3 post-transplantation years of pregnant recipients. Their findings suggested that pregnancy within 2 post-transplantation years was significantly related to abortion and elevated allograft loss.^14^ In addition, other investigations demonstrated that pregnancy should not be planned within the first year of transplantation to allow stabilization of allograft function and also reduction of immunosuppressant dose to maintenance levels.^15^

No functional decline or estimated glomerular filtration rate (eGFR) reduction was observed in the present case; however, patients after kidney transplantation with single kidneys are at higher risk of eGFR abnormalities. In this regard, Park et al indicated an adverse pregnancy outcome in pregnant women with abnormal eGFR during pregnancy.^16^ However, Bramham et al reported that there is not any significant correlation between low eGFR levels in post-transplanted pregnant women and poor pregnancy outcomes.^17^

Regarding the delivery modes, vaginal delivery was considered to be the safest method of delivery in women with heart-kidney transplantation. The cesarean method should be considered for the obstetrical indications^18^ that were performed in the present case based on the obstetric and gynecologic consultations.

## Conclusion

This case was the first successful pregnancy and delivery by a heart-kidney transplanted patient in Iran. The successful outcome of pregnancy in this woman could be due to the proper cooperation between the medical team and patient, as well as the appropriate timing of pregnancy.

Finally, we can conclude that post-transplantation pregnancy can be safe with some consideration and caution.

## Patient consent statement

The authors received all necessary patient consent with regards to the publication of this case report.

## Disclosure statement

The authors declare that they have no known competing financial interests or personal relationships that could have appeared to influence the work reported in this paper.
